# From L-Dopa to Dihydroxyphenylacetaldehyde: A Toxic Biochemical Pathway Plays a Vital Physiological Function in Insects

**DOI:** 10.1371/journal.pone.0016124

**Published:** 2011-01-24

**Authors:** Christopher Vavricka, Qian Han, Yongping Huang, Sara M. Erickson, Kim Harich, Bruce M. Christensen, Jianyong Li

**Affiliations:** 1 Department of Biochemistry, Virginia Tech, Blacksburg, Virginia, United States of America; 2 Institute of Plant Physiology and Ecology, Shanghai Institutes for Biological Sciences, Shanghai, China; 3 Department of Pathobiology, University of Wisconsin-Madison, Madison, Wisconsin, United States of America; The George Washington University, United States

## Abstract

One protein in *Aedes aegypti*, classified into the aromatic amino acid decarboxylase (AAAD) family based on extremely high sequence homology (∼70%) with dopa decarboxylase (Ddc), was biochemically investigated. Our data revealed that this predicted AAAD protein use L-dopa as a substrate, as does Ddc, but it catalyzes the production of 3,4-dihydroxylphenylacetaldehyde (DHPAA) directly from L-dopa and apparently has nothing to do with the production of any aromatic amine. The protein is therefore named DHPAA synthase. This subsequently led to the identification of the same enzyme in *Drosophila melanogaster*, *Anopheles gambiae* and *Culex quinquefasciatus* by an initial prediction of putative DHPAA synthase based on sequence homology and subsequent verification of DHPAA synthase identity through protein expression and activity assays. DHPAA is highly toxic because its aldehyde group readily reacts with the primary amino groups of proteins, leading to protein crosslinking and inactivation. It has previously been demonstrated by several research groups that *Drosophila* DHPAA synthase was expressed in tissues that produce cuticle materials and apparent defects in regions of colorless, flexible cuticular structures have been observed in its gene mutants. The presence of free amino groups in proteins, the high reactivity of DHPAA with the free amino groups, and the genetically ascertained function of the *Drosophila* DHPAA synthase in the formation of colorless, flexible cuticle, when taken together, suggest that mosquito and *Drosophila* DHPAA synthases are involved in the formation of flexible cuticle through their reactive DHPAA-mediated protein crosslinking reactions. Our data illustrate how a seemingly highly toxic pathway can serve for an important physiological function in insects.

## Introduction

It has long been recognized that insect cuticle contributes substantially to their evolutionary success. The importance of cuticle for insect survival also has made it one of the primary targets for insect pest and disease vector control [1], [2]. Insect cuticle is 1) primarily composed of chitin, proteins and lipids, 2) formed multiple times throughout development, and 3) found in different forms throughout the insect body. The cuticular exoskeleton provides insects with protection against physical injury and infection, rigidity for muscle attachment and mechanical support and flexibility for joint movement. During development and growth, insects have to periodically shed their old cuticle and produce a new one. The newly formed cuticle is soft, which allows it to stretch and expand to accommodate the increased body size and changed body shape. The newly formed cuticle is vulnerable to adverse environmental conditions and must be promptly hardened or solidified shortly after insects shed their old cuticle. Cuticle protein crosslinking is a key biochemical event involved in cuticle hardening or sclerotization.

The major insect cuticular types include the rigid/hard (e.g., sternite or tergal plates) and flexible, membranous cuticle (e.g., joint regions in appendages and between cuticular plates). The drastically different physical properties of these cuticle types are a result of various chitin and protein compositions [3], [4], as well as the abundance and type of sclerotization or protein cross-linking occurring within [5], [6]. Two sclerotization precursors, *N*-acetyldopamine (NADA) and *N*-β-alanyldopamine (NBAD) are synthesized from dopamine, in a pathway starting with tyrosine, and including tyrosine hydroxylase and L-dopa decarboxylase (Ddc) enzymes among others downstream. Both NADA and NBAD can be oxidized to *ortho*-quinones and *para*-quinone methides which produce mono-adducts via a ring-position or β-position (side-chain) covalent bond, respectively. Many insect cuticles undergo darkening or tanning in combination with sclerotization. This process involves the formation of melanin from cuticular dopamine, which can be incorporated into the cuticular protein matrix via the NADA and NBAD crosslinkers [5]. It is predicted that a number of processes, each with inherent complexities, could be involved in the optimal sclerotization of various insect cuticle types [6].

Dopamine occupies a special position in insect cuticle melanization/sclerotization: its acetylation or β-alanine conjugation results in the formation of sclerotization precursors and its oxidation leads to cuticle melanization. Consequently, Ddc involved in dopamine production has attracted considerable attention. Ddc is commonly named aromatic amino acid decarboxylase (AAAD) in many species. As more completely sequenced insect genomes are available, it becomes clear that insects have more Ddc-like or AAAD sequences than non-insect species. For example, the *Aedes aegypti* genome reveals that this insect contains seven AAAD sequences with high sequence similarities. Among them, its Ddc is the prototype and well-characterized, and other *Ae. aegypti* AAAD sequences have been named based on their high sequence homology with Ddc. Mosquitoes are a well studied insect family because of the role many species play in transmitting infectious disease pathogens during blood feeding on vertebrates. The yellow fever mosquito (*Aedes aegypti*) is a species frequently studied, and is one of three mosquitoes with a completely sequenced genome [7].

Herein, we investigated one *Ae. aegypti* protein (NCBI protein ID: EAT37247) we predicted was involved in dopamine production as Ddc. It is classified as an AAAD protein in VectorBase (http://www.vectorbase.org) based on its high sequence homology (70%) with Ddc. Our biochemical studies, however, led to a discovery that EAT37246 recombinant protein catalyzes the production of 3,4-dihydroxyphenylacetaldehyde (DHPAA) directly from L-dopa and is not involved in dopamine production.

## Results

### Expression of recombinant *Ae. aegypti* Ddc and EAT37246 proteins

**Figure 1:**
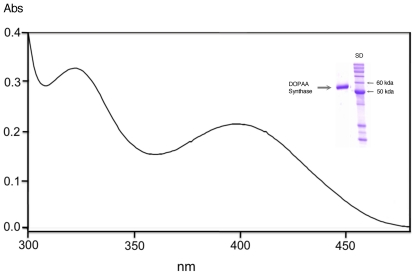
Spectral characteristics of *Ae. aegypti* EAT37246 recombinant protein. Purified protein was prepared in 50 mM phosphate buffer (pH 7.5) and its absorbance spectrum was determined using a Hitachi U2001 UV-Visible spectrophotometer. Insert illustrates purified protein and reference molecular weight markers.

The NCBI sequences with protein ID of EAT37246 & EAT37247 correspond to *Ae. aegypti* AAEL010735 & AAEL010735 in VectorBase, respectively. For consistency, their protein IDs are used throughout. The high sequence homology of the deduced sequence of EAT37246 with Ddc (Fig. S1, supplement) suggested that this protein is involved in the production of aromatic amines. To compare its functional similarities with Ddc, the EAT37246 sequence was expressed along with *Ae. aegypti* Ddc (NCBI protein ID: EAT33489) using a bacterial protein expression system. The EAT37246 recombinant protein displayed visible absorbance peaks (Fig. 1) similar to that observed in Ddc (not shown), indicating that it contained the pyridoxal 5-phosphate (PLP) co-factor.

### Substrate screening

**Figure 2:**
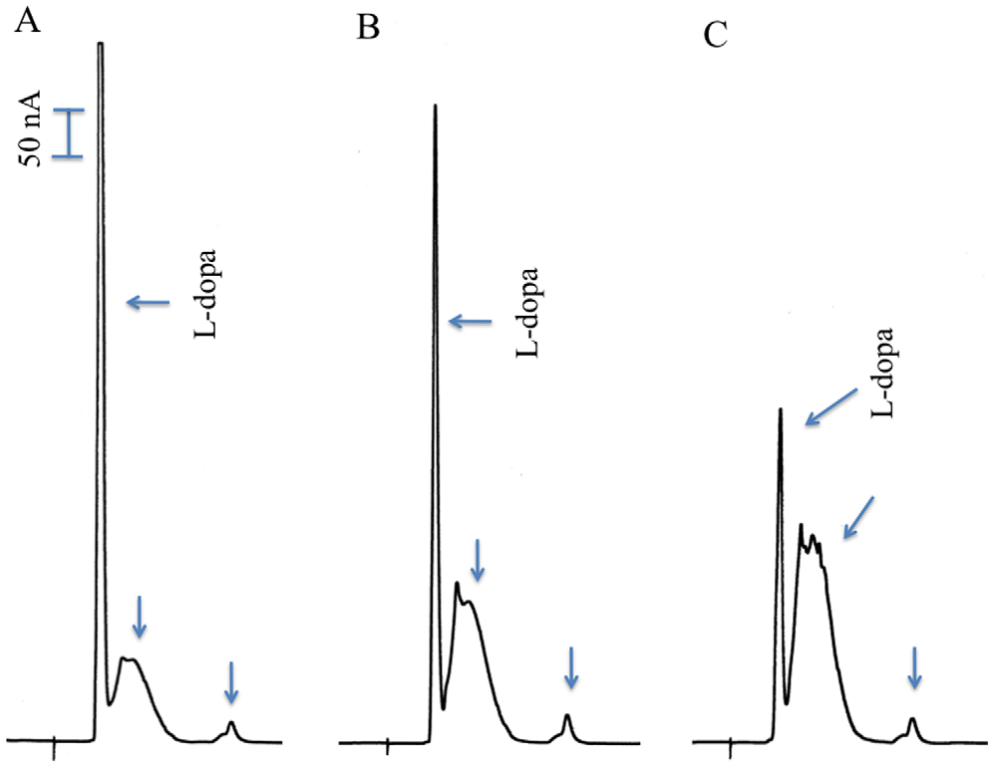
Activity of recombinant EAT37246 protein to L-dopa. Chromatograms A, B, and C illustrate L-dopa remained and product formed (arrow pointed peaks) in L-dopa and recombinant EAT37246 protein reaction mixture at 15 min (A), 30 min (B) and 45 min (C) after incubation, respectively. The total volume of the reaction mixture was 100 µl, the amount of recombinant protein incorporated into the reaction mixture was 25 µg and the final concentration of L-dopa in the reaction mixture was 2 mM. The reaction mixtures were incubated at 25°C.

Purified EAT37246 recombinant protein was screened against the aromatic amino acids L-histidine, L-phenylalanine, L-tyrosine, L-dopa, and L-tryptophan. No product was detected in the reaction mixtures containing any aromatic amino acids except L-dopa, where a broad peak and a very minor peak were observed in the reaction mixtures (Fig. 2A). This broad peak was initially considered to be contaminates from substrate or nonenzymatic oxidation products from L-dopa. As incubation continued, the L-dopa peak decreased in concert with a concomitant increase of the broad peak (Fig. 2B & 2C), indicating that the broad peak was not contaminant of the substrate. The broad peak was not observed when D-dopa was used in the reaction mixtures (not shown), also excluding the possibility of the broad peak being a nonenzymatic oxidation product of L-dopa. When Ddc recombinant protein was mixed with L-dopa, L-dopa was rapidly converted to dopamine and the broad peak was never observed even after an extended incubation period (not shown). These results demonstrate that EAT37246 recombinant protein uses L-dopa as its substrate, similar to Ddc, but its primary product is very different.

### Product identification

The common reaction mechanism of PLP-dependant enzymes likely applies to the EAT37246 recombinant protein and its L-dopa substrate. Accordingly, changes of L-dopa are probably limited to its carboxyl group, amino group or both. The very minor peak was identified as dopamine, which could mean that L-dopa was first decarboxylated to dopamine and dopamine was then converted to a product that behaves as a broad peak during chromatography. However, incubation of dopamine with EAT37246 recombinant protein did not result in the detection of the broad peak (not shown). When 3,4-dihydroxypyruvate (product of L-dopa half transamination) was analyzed under identical conditions, the compound eluted as a sharp peak and did not co-elute with the broad peak (not shown), eliminating the possibility of the broad peak as the product of L-dopa half transamination.

**Figure 3:**
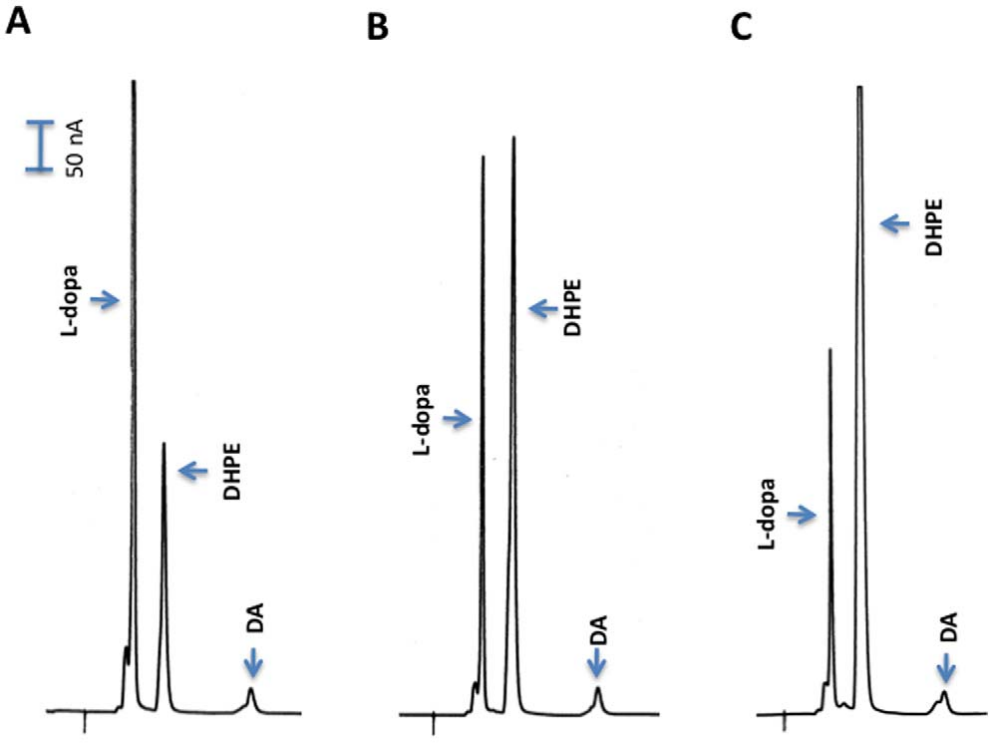
Product identification. Reaction mixture (100 µl) was prepared as described in Figure 2. At 15, 30 and 45 min after incubation, 16 µl of the reaction mixture was withdrawn and mixed with 4 µl ethanol saturated with NaBH_4_. The mixtures (now 20 µl) were incubated at room temperature for 5 min and then 20 µl of 0.6 M formic acid was incorporated into the mixtures (to decompose remaining NaBH_4_). After centrifugation, the acidified reaction mixtures were analyzed by HPLC-ED. Chromatograms A, B and C correspond to borohydride-treated reaction mixtures that had been incubated at 25°C for 15, 30 and 45 min prior to borohydride treatment, respectively. Note that these chromatograms should closely reflect the chromatograms in Figure 2A–2C, except that the compound corresponding to the broad peak has been converted to DHPE (3,4-dihydroxyphenylethanol) through borohydride reduction (please see diagram 1 for detail). The DHPE formed through borohydride reduction has been further confirmed by comparison with authentic DHPE standard.

**Figure 4:**
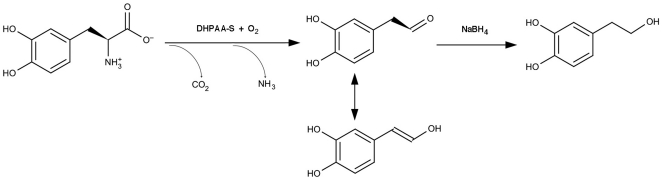
DHPAA synthase-catalyzed L-dopa to DHPAA pathway. DHPAA synthase catalyzes both decarboxylation and deamination of L-dopa to DHPAA. DHPAA might undergo dynamic changes between enol-keto isomers under weak acidic condition, which might explain in part the broadness of the DHPAA peak during HPLC-ED analysis. DHPAA is converted to 3-dihydroxyphenylethanol in the presence of NaBH_4_.

**Figure 5:**
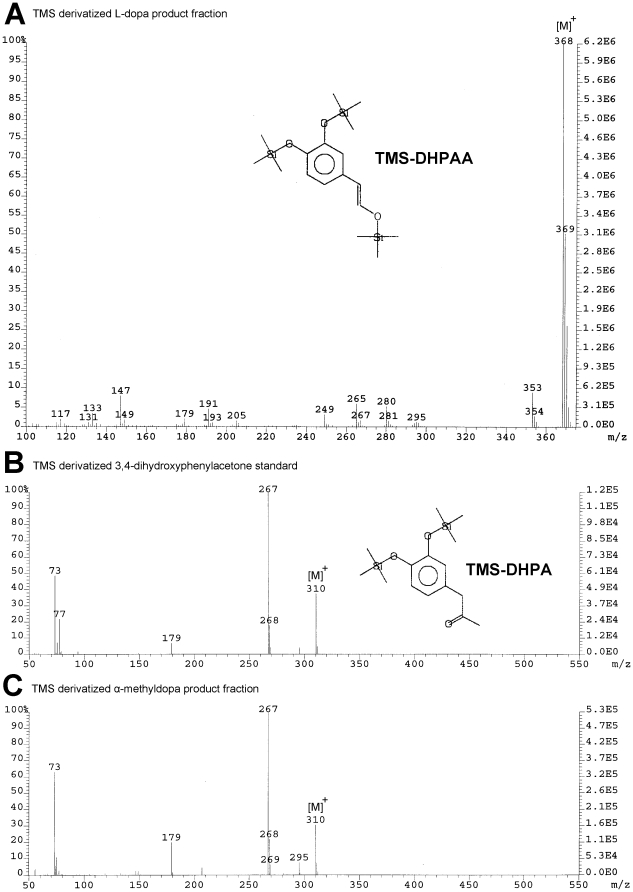
Analysis of DHPAA-mediated reaction products by GC/MS. GC/MS electron impact fragmentation spectrum of the trimethylsilyl (TMS) derivatized broad peak from *Ae. aegypti* DHPAA synthase (AMD-r protein) and L-dopa reaction mixture (A), electron impact fragmentation spectrum of the TMS derivatized product from *Ae. aegypti* DHPAA synthase and AMD reaction mixture (B), and electron impact fragmentation spectrum of the TMS derivatized AMD standard (C).

It also is possible that L-dopa might first undergo decarboxylation, but the resulting dopamine is not released until it is oxidatively deaminated, leading to the production of 3,4-dihydroxyphenyl acetaldehyde (DHPAA). After the EAT37246 recombinant protein and L-dopa reaction mixtures were incubated and treated with sodium borohydride (NaBH_4_), followed by HPLC-ED analysis under the same conditions, the broad peak was converted to a very sharp peak (Fig. 3A–3C). Aldehyde and keto groups can be reduced to alcohol groups by NaBH_4_, which reduces DHPAA (if formed) to 3,4-dihydrophenylethanol (DHPE). Comparison of authentic DHPE standard with NaBH_4_-treated reaction mixtures determined that DHPE co-eluted with the NaBH_4_ reduced peak at different conditions of HPLC-ED analysis (not shown). Under weak acidic conditions during HPLC-ED analysis, the aldehyde group of DHPAA might undergo dynamic changes between enol-keto isomers, which might explain in part the broadness of the DHPAA peak (Fig. 4). These data provided sufficient evidence for the identification of DHPAA as the broad peak formed in the EAT37246 recombinant protein and L-dopa reaction mixture. The identity of the product as DHPAA was further verified by its mass spectrum by GC/MS (Fig. 5A). Based on its enzymatic product, we named EAT37246 DHPAA synthase. In *Ae. aegypti*, the EAT37247 and EAT37246 are encoded by two different genes, but their primary sequences are essentially the same except that residue Asn235 in EAT37246 is replaced by Tyr235 in EAT37247. Their extremely high sequence identity provides sufficient data to conclude that EAT37247 also is DHPAA synthase.

### Reactivity of DHPAA

**Figure 6:**
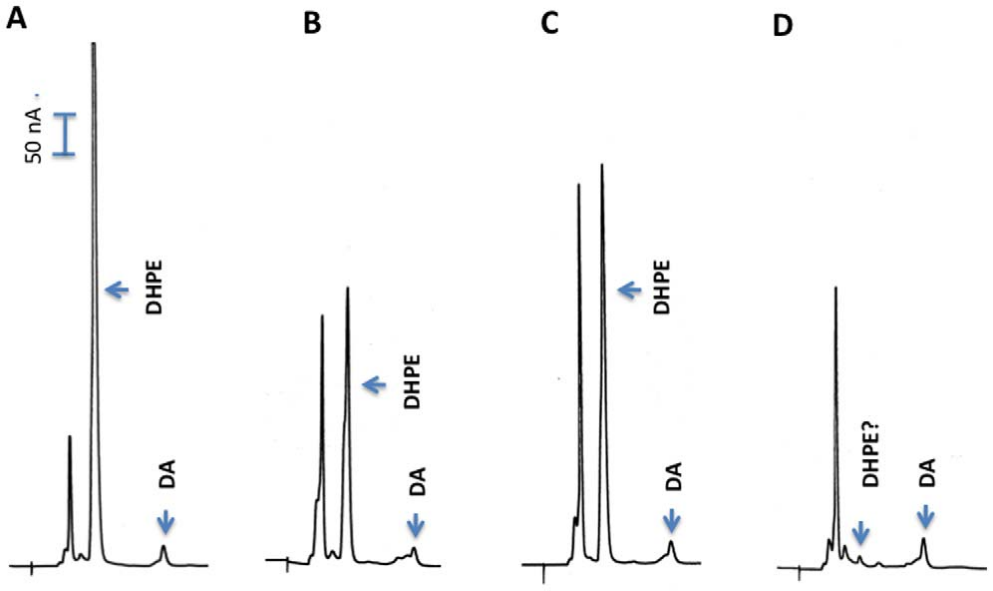
Instability and reactivity of DHPAA. DHPAA synthase and L-dopa reaction mixtures were prepared as in Figures 2 and 3, but some reaction mixtures also contained 5 mM Nα-acetyl-lysine or Nα-acetyl-lysine methyl ester. Chromatograms **A** and **B** illustrate the relative amounts of DHPAA in the DHPAA synthase and L-dopa reaction mixture in 50 min incubation in absence and presence of 5 mM Nα-acetyl-lysine, respectively. Chromatograms **C** and **D** show the relative amount of DHPAA in the reaction mixture at 3.0 hr incubation in absence and presence of 5 mM Nα-acetyl-lysine, respectively. The reaction mixtures were treated NaBH_4_ prior to HPLC-ED analysis, which converts DHPAA to DHPE. The relative amounts of DHPE reflect the remaining DHPAA in these reaction mixtures.

DHPAA appears to be highly reactive based on its chromatographic behavior (see Fig. 2 & Fig. 4). After the majority (>90%) of L-dopa has been converted to DHPAA, progressive decay of the product was observed. For example, approximately 35–40% of the DHPAA remained in the reaction mixture for 3 hr at room temperature (Fig. 6A & 6C). When Nα-acetyl-lysine or Nα-acetyl-lysine methyl ester was also present in the EAT37246 recombinant protein and L-dopa reaction mixtures, accumulation of DHPAA was decreased noticeably (Fig. 6B) and only a trace amount of DHPAA remained in the reaction mixture after 3 hr incubation at room temperature (Fig. 6D). The rapid decrease of DHPAA in the reaction mixtures in the presence of Nα-acetyl-lysine suggests that DHPAA reacts with the lysine derivative, leading to the formation of DHPAA-Nα-acetyl-lysine complex. However, DHPAA-Nα-acetyl-lysine complex was not detected during HPLC-ED analysis, which likely is due to the inability of the working electrode to oxidize the complex at the applied conditions.

### *Drosophila* α-methyl dopa resistant (AMD-r) gene encoding for DHPAA synthase

**Figure 7:**
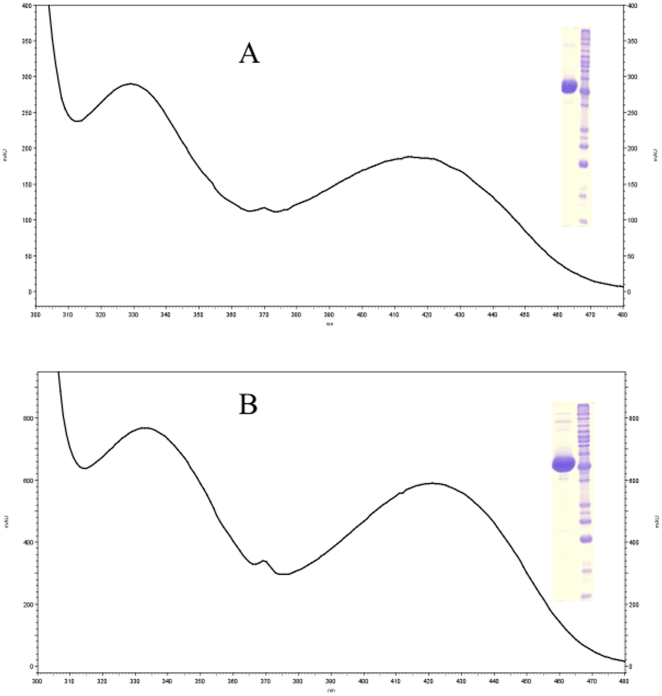
Spectral characteristics of *D. melanogaster* DHPAA synthases. Purified NP_724162 recombinant protein and NP_476592 recombinant protein were prepared in 50 mM phosphate buffer (pH 7.5) and their absorbance spectrum was determined using a Hitachi U2001 UV-Visible spectrophotometer. Spectra A and B illustrate the spectral characteristics of *D. melanogaster* NP_724162 recombinant protein and NP_476592 recombinant protein, respectively. Insert shows purified protein and reference molecular weight markers.

**Figure 8:**
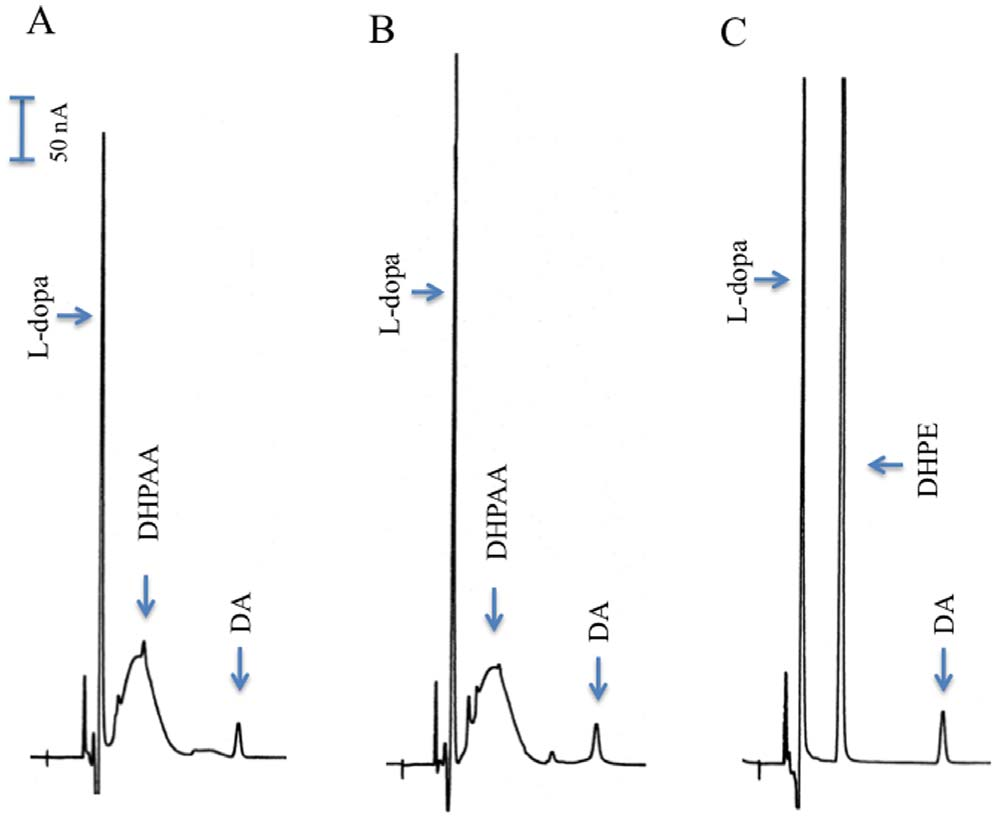
DHPAA synthase activity of *Drosophila* AMD-r proteins. Chromatograms illustrate DHPAA formed in a L-dopa and recombinant NP_476592 protein reaction mixture (A) and a L-dopa and recombinant NP_724162 reaction mixture (B) at 25 min after incubation, respectively. The total volume of the reaction mixture was 100 µl, the amount of recombinant protein incorporated into the reaction mixture was 25 µg and the final concentration of L-dopa in the reaction mixture was 2 mM. The reaction mixtures were incubated at 25°C. Chromatogram (C) shows conversion of DHPAA to 3,4-dihydroxyphenylethanol (DHPE) by NaBH_4_ in a L-dopa and recombinant NP_724162 reaction mixture (prepared and analyzed as those described in Figure 3).

A BLAST search of mosquito DHPAA synthase against the *D. melanogaster* database identified that its AMD-r proteins (NP_476592 and NP_724162) share a slightly, but noticeably better sequence identity (53–56%) than that (50%) of *Drosophila* Ddc with the mosquito DHPAA synthase. After both AMD-r isoforms were expressed (Table S1) and their recombinant proteins were analyzed, we found that the spectrum of both AMD-r proteins (Fig. 7) in the visible region is similar to that of *Ae. aegypti* DHPPA synthase and both have the same activity (Fig. 8) as that of mosquito DHPAA synthase.

### Activity of DHPAA synthase to α-methyl dopa (AMD)

**Figure 9:**
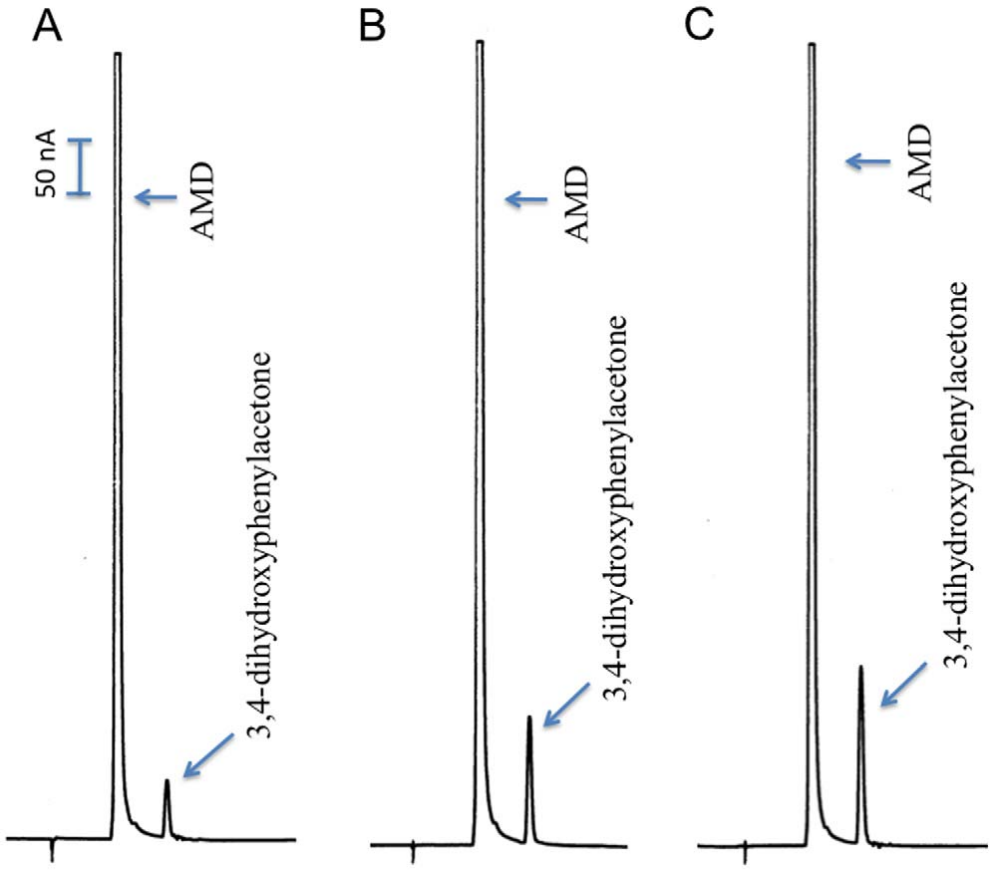
Production of 3,4-dihydroxyphenylacetone from AMD by AMD-r protein. Reaction mixture (100 µl) was prepared as described in Figure 2 (except 25 µg of AMD-r protein, expressed using CDS of the NP_476592 sequence, were used). At 15, 30 and 45 min after incubation, 20 µl of the reaction mixture was withdrawn and mixed with an equal volume of 0.6 M formic acid. The mixture was centrifuged and supernatant was analyzed by HPLC-ED. Chromatograms A, B and C illustrate the relative amounts of 3,4-dihydrophenylacetone (arrow pointed) formed during 15, 30 and 45 min incubation, respectively.

**Figure 10:**
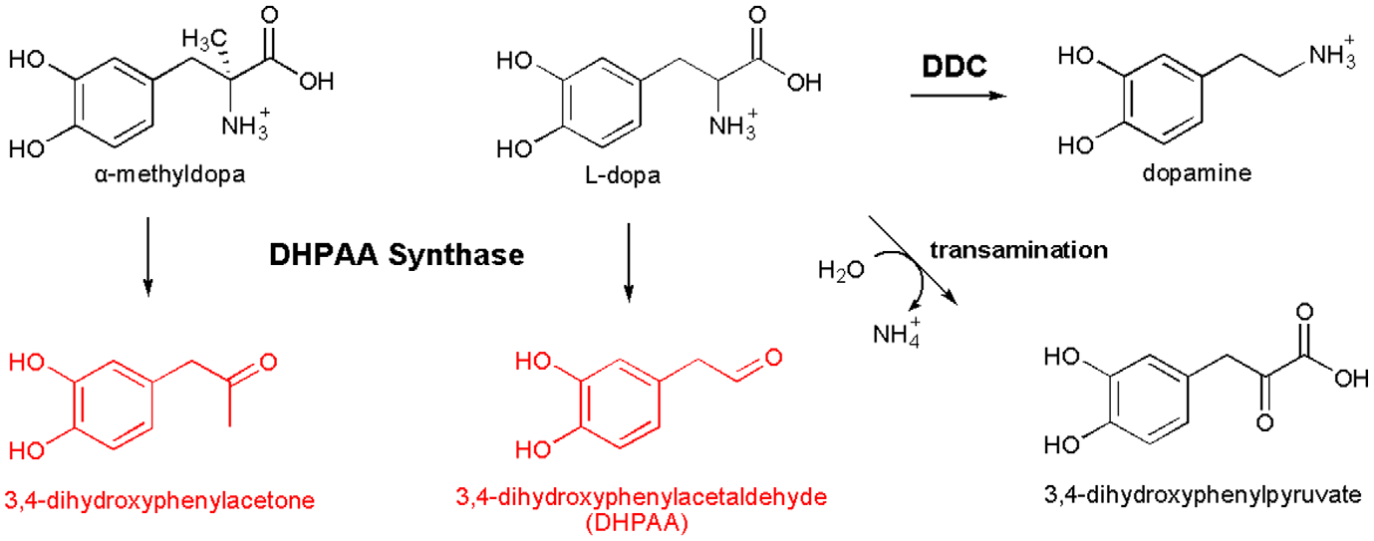
Comparison of DHPAA synthase-mediated reactions, Ddc-catalyzed reaction and L-dopa half transamination reaction. DHPAA synthase catalyzes a complicated decarboxylation-deamination process of L-dopa and AMD, leading to the production of DHPAA and 3,4-dihydroxyphenylacetone, respectively, which contrasts to Ddc-catalyzes decarboxylation of L-dopa to dopamine and half-transamination of L-dopa to 3,4-dihydroxyphenylpyruve.

When *Drosophila* AMD-r (DHPAA synthase) recombinant proteins were mixed with AMD, progressive accumulation of a product in the reaction mixtures was observed (Fig. 9), but the reaction proceeds slowly (about 10–12% of its activity to L-dopa). GC/MS analysis identified the product as 3,4-dihydroxyphenylacetone (Fig. 5B & 5C). In addition, 3,4-dihydroxyphenylacetone displayed as a sharp peak during HPLC-ED analysis. Obviously the α-methyl group has a considerable stabilizing effect on the enzymatic product (the carbonyl carbon is less positive due to the presence of the electron donating methyl group). When the recombinant mosquito DHPAA synthase was mixed with AMD, the same product was detected (not shown). Figure 10 illustrates the reactions mediated by DHPAA synthase (AMD-r protein) with L-dopa and AMD as substrates and its difference with the Ddc-catalyzed reaction and the half-transamination reaction of L-dopa to 3,4-dihydroxyphenylpyruvate. The ability to use AMD as a substrate may explain the observed function of DHPAA synthase in relation to AMD resistance.

### DHPAA synthase in other *Drosophila* and mosquito species

**Figure 11:**
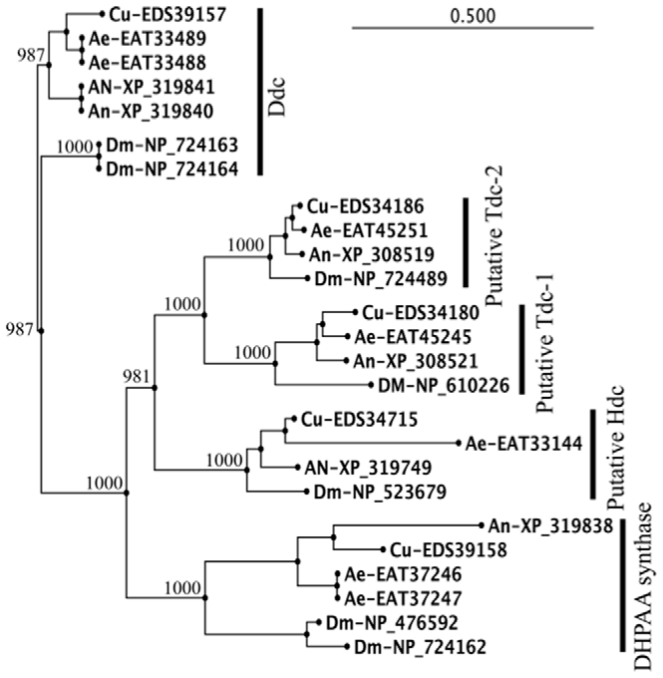
Phylogenetic analysis of mosquito and *Drosophila* DHPAA synthases with other insect AAAD sequences. The overall sequence identity among the compared sequences ranged from 32–100%. Sequence identity of individual groups: Ddc group, 74–100%; Putative Hdc group, 69–90%; putative Tdc-1 group, 63–87%; putative Tdc-2 group, 76–94%; DHPAA synthase group, 53–100%. The protein sequences were aligned with CLUSTAL W (ver. 2.0.10) and the alignment was manually checked. Then the phylogenetic and molecular evolutionary analyses were conducted using the neighbor-joining method with 1000 times bootstrap embedded in the CLC Main Workbench software (ver. 5.7.1). Abbreviations: Ae, *Aedes aegypti*; Cu, *Culex quinquefasciatus*; An, *Anopheles gambiae*, Dm, *Drosophila melanogaster*; Ddc, L-dopa decarboxylase; Tdc, L-tyrosine decarboxylase.

A BLAST search of NCBI *Drosophila* and mosquito species databases identified numerous protein sequences with identity at ≥50% identity with *Ae. aegypti* DHPAA synthase. These sequences either have been named AAAD, Ddc-like proteins, aspartate aminotransferase superfamily proteins or α-methyldopa resistant (AMD-r) proteins. Among the other 12 available *Drosophila* genomes, each of them contains at least one AMD-r protein with sequence identity ranged from 70–99% to that of the functionally verified *D. melanogaster* DHPAA synthases (or AMD-r proteins). The *Anopheles gambiae* XP_319838 (AGAP009090 in VectorBase) and *Culex quinquefasciatus* EDS39158 (CPIJ013308 in VectorBase) share the highest sequence identity (83–86%) with *Ae. aegypti* DHPAA synthase, but the coding sequence (CDS) of the *An. gambiae* XP_319838 is missing a 5′-end exon and a 3′-end exon and the CDS of the *Cu. quinquefasciatus* EDS39158 is missing a 5′-end exon. Based on genomic data in VectorBase, their entire CDS was reassembled, PCR amplified and the sequences verified (Fig. S2). Interestingly, the *Cu. quinquefasciatus* EDS39158 sequence has been named AMD-r protein, which apparently was based on its slightly higher sequence identity (52%) with *D. melanogaster* AMD-r protein than with that (45–46%) of *Drosophila* and mosquito Ddc (Fig. 11). The DHPAA synthase identity of the *An. gambiae* XP_319838 and *Cu. quinquefasciatus* EDS39158 sequences was determined by subsequent expression (Table S1) and biochemical analysis of their recombinant proteins (not shown). The biochemical verification of *D. melanogaster* AMD-r proteins (NP_476592 and NP_724162) as DHPAA synthases and the presence of AMD-r protein in each of other 12 sequenced *Drosophila* species, along with DHPAA synthases in three mosquito genera, provides the basis to predict that at least one DHPAA synthase is present in different mosquitoes and *Drosophila* species.

## Discussion

The high sequence identity that DHPAA synthase shares with Ddc (Fig. S1) could have easily led to the assumption that DHPAA synthase plays a role in aromatic amine production similar to that of Ddc. This likely has been the major impediment to the discovery of its true function. The *Ae. aegypti* DHPAA synthase proteins had been classified as AAAD based on their high sequence identity with Ddc Similarly the AMD-r protein could have been called Ddc isozymes, if not for its phenotypic association with AMD resistance [8]–[16]. Even with its AMD-resistant phenotype, the assigned functions and biochemical processes of the AMD-r gene in the FlyBase have apparently been based on those of *Drosophila* Ddc. For example, the molecular functions of the AMD-r gene are described in FlyBase as “aromatic L-amino acid decarboxylase activity and pyridoxal phosphate binding” and its biological process involvement being “catecholamine metabolic process, chitin-based cuticle development, dopamine metabolic process; cellular amino acid and derivative metabolic process; and carboxylic acid metabolic process” (http://flybase.org/reports/FBgn0000075.html). Based on its biochemical activity, these described functions and biochemical processes of the AMD-r gene in the FlyBase either are wrong, not relevant or too vague to be physiologically meaningful. Our identification of AMD-r protein as DHPAA synthase and its biochemical difference highlight the fact that sequences sharing high identity may have very different biochemical functions.

There are no reports of DHPAA being a natural metabolite in insects. In mammals, DHPAA is produced from dopamine by monoamine oxidase (MAO)-catalyzed reaction and is considered a pathway used to detoxify excessive dopamine. However, DHPAA is unstable and readily reacts with primary amines, leading to protein crosslinking/inactivation [17]. To counteract this, mammals use a number of aldehyde dehydrogenases to rapidly detoxify DHPAA to 3,4-dihydroxyphenylacetate [18]. Even so, DHPAA has been linked to the development of a number of neurodegenerative diseases [18]–[23] and human MAO proteins have been the major targets for the development of inhibitors [24]–[27]. Overall, the current literature suggests the toxicity of the dopamine to DHPAA pathway outweighs its benefits. Because DHPAA readily crosslinks and inactivates proteins, it would be highly toxic if allowed to freely circulate in the open circulatory system of mosquitoes. This leads to the critical question as to why these enzymes have evolved in mosquitoes and/or insects.

The determination of the one *Ae. aegypti* AAAD protein in DHPAA synthesis further led to the unambiguous identification of the same DHPAA synthesizing enzymes from several other insect species. A BLAST search of the mosquito DHPAA synthase against the NCBI NR protein database identified dozens of insect proteins sharing high sequence homology with DHPAA synthase. These proteins are designated AAAD proteins, Ddc proteins or α-methyldopa resistant (AMD-r) proteins. Among them, AMD-r proteins share slightly better (about 3–5%) sequence identity with mosquito DHPAA synthase than Ddc proteins. AMD-r proteins were named based on observations that *D. melanogaster* with a mutation of its CG10501 gene became sensitive to α-methyldopa (AMD) added to their food [8]–[16]. Homozygous *Drosophila* AMD-r gene mutants die during embryonic development [11]–[13], indicating that the function of AMD-r gene is essential. The AMD-r gene is expressed in tissues that produce cuticle and noticeable defects in regions of flexible cuticular structures have been observed in AMD-r gene mutants [14]–[16], but the biochemical mechanism/pathways involved in flexible cuticle formation are unknown.

We hypothesize that *Drosophila* AMD-r proteins are DHPAA synthases and our subsequent characterization of two *D. melanogaster* recombinant AMD-r proteins indeed demonstrate that AMD-r proteins have the same activity as mosquito DHPAA synthase. Our discovery of the biochemical action of mosquito DHPAA synthase, in conjunction with the high reactivity of its enzymatic product in protein crosslinking, and the genetic evidence of a role for AMD-r gene (or DHPAA synthase gene) in flexible cuticle formation [14]–[16], when taken together, suggests that DHPAA synthase is involved in flexible cuticle formation through its reactive product-mediated protein crosslinking reactions (our recent analysis of DHPAA synthase and L-dopa reaction mixtures in the presence of lysine containing peptides by mass spectrometry suggests that DHPAA interacts with lysine to form complexes, but we need more time to clearly establish the overall mechanisms and structures of the complexes).

Although formation of a hardened cuticle is vital for the survival of insects, if the entire cuticle were hardened uniformly, insects would be immobile. Consequently, some areas of the insect cuticle must be rigid enough to maintain the body shape and support muscle attachment, while other areas must be flexible to allow for body movement and/or expansion. Cuticle melanization often is closely related to the formation of stiff cuticular structures, e.g., the strong, rigid mandible that often is heavily melanized in many insect species. In contrast, unpigmented or colorless areas often correspond to flexible cuticle structures, which seems particularly true in most Dipterans. There have been numerous studies dealing with the biochemical reactions and mechanisms involved in cuticle hardening and melanization, but there has been limited discussion regarding the regulation and biochemical processes specific for the formation of unpigmented/flexible, yet highly protective cuticle structures. Based on the defect of unpigmented, flexible cuticle structures in *Drosophila* AMD-r gene mutants [14]–[16], we believe that in *Drosophila* and mosquitoes (possibly many other insect species as well) DHPAA synthase plays an essential role in the formation of flexible cuticle structures through promoting cuticle protein crosslinking by its reactive product and minimizing Ddc-mediated cuticle sclerototization (by NADA and NBAD) and melanization by channeling L-dopa to DHPAA.

In summary, this study provides data that demonstrate the biochemical function of one *Ae. aegypti* AAAD sequence as DHPAA synthase and revealed that *Drosophila* AMD-r proteins as its DHPAA synthases. Through sequence comparison of the functionally determined DHPAA synthase proteins, we are able to establish that at least one DHPAA synthase is present in other *Drosophila* and mosquito species. The presence of free amino groups in proteins, the high reactivity of DHPAA with the free amino groups, and the genetically ascertained function of the *Drosophila* AMD-r gene (its DHPAA synthase gene) in the formation of colorless, flexible cuticle [14]–[16], when taken together, indicate that mosquito and *Drosophila* DHPAA synthase is involved in the formation of flexible cuticle through its reactive DHPAA-mediated protein crosslinking reactions. Although it is far from a comprehensive understanding in terms of the detailed biochemical processes, pathways, and chemical mechanisms of DHPAA synthase-mediated flexible insect cuticle formation, data present in this communication are highly significant in that they provide practical insights into designing experiments that will lead to a comprehensive understanding of the DHPAA synthase in insect cuticle formation.

## Materials and Methods

### Reagents

L-dopa, dopamine, AMD, PLP, pyridine, sodium phosphate, formic acid, and acetonitrile were from Sigma (St. Louis, MO). Trimethylchlorosilane (TMS) and N,O-Bis(trimethylsilyl)trifluoroacetamide (BSTFA) were obtained from Pierce (Waltham, MA). The IMPACT-CN protein expression system was purchased from New England Biolabs (Ipswich, MA).

### Protein expression and purification

All proteins were expressed and purified according to the previous methods used in *D. melanogaster* Ddc recombinant production [28] with modifications. *Ae. aegypti* EAT37246 was first expressed and purified. Subsequently, recombinant proteins corresponding to *D. melanogaster* AMD-r proteins (NP_476592 & NP_724164), *An. gambiae* XP_319838 (AGAP009090 in VectorBase) and *Cu. quinquefasciatus* EDS39158 (CPIJ013308 in VectorBase) were expressed and purified (Table S1, supplement, provided all primer pairs utilized for the amplification of *Drosophila* and mosquito DHPAA synthase coding sequences).

### Product identification

The identification of the primary product by EAT37246 recombinant protein to L-dopa was through systematic elimination of possible decarboxylation product or deamination product and analysis of NaBH_4_ treated reaction mixtures in comparison with the predicted authentic compound as described in results section. The identity of the product was further confirmed by GC/MS analysis (Fig. 5A).

### DHPAA synthase activity assay

A typical reaction mixture of 100 µl containing 25 µg mosquito or *Drosophila* recombinant DHPAA synthase and 2 mM of L-dopa or AMD was prepared in 50 mM phosphate buffer (pH 6.8). The reaction mixtures were incubated at 25°C in a water bath. At 15, 30 and 45 min intervals, 20 µl was withdrawn from each reaction mixture and mixed with an equal volume of 0.8 M formic acid to stop the reaction. The acidified reaction mixtures were centrifuged and supernatants (5 µl) were analyzed by reverse-phase HPLC with electrochemical detection (HPLC-ED) or treated with NaBH_4_ before HPLC-ED analysis. The mobile phase consisted of 50 mM citrate buffer (pH 3.2) containing 10% (v/v) acetonitrile and 1 mM octyl sulfate for the analysis of reaction mixtures with dopa as a substrate or 20% acetonitrile with AMD as a substrate.

### Identification of DHPAA synthase enzymatic products using GC/MS

Fractions, corresponding to the broad product peak and sharp product peak in DHPAA synthase and L-dopa and DHPAA synthase and AMD reaction mixtures respectively, were collected and TMS derivatized for GC/MS analysis [29]. GC/MS analyses were performed using a Hewlett-Packard 5890 series gas chromatographic system interfaced to a VG 70S mass spectrometer equipped with an Opus 3.1 data system. Chromatographic separations were achieved using a RTX5MS capillary column (30 M, 0.32 mM i.d., 0.25 µM film thickness, Restek). Helium carrier gas was employed. Oven temperature was programmed from 80°C (for 1 minute) to 280°C at 8°C/min. Injector temperature was 225°C and the interface line was 250°C. Injections of 2 to 5 µL were performed in the splitless mode. Electron impact ionization mass spectra were obtained at an electron energy of 70 eV, a trap current of 200 µA, an acceleration voltage of 8 kV and a resolution of 1000 (10% valley definition). The mass spectrometer was scanned at 1 second/decade over the range of m/z 50–550. The temperature of the ion source was 200°C. Enzymatic product electron impact fragmentation spectra were compared to corresponding standard spectra. A DHPAA standard spectrum from Mattammal et al. was used [30] and a 3,4-dihydroxyphenylacetone standard was synthesized according to the methods of Slates et al [31].

## Supporting Information

Figure S1 Sequence comparison of *Ae. aegypti* DHPAA synthase and Ddc. Letters with magenta and cyan background are fully conserved and strongly conserved amino acid residues, respectively. Lys306 in DHPAA-S and Lys303 (yellow background) in Ddc are involved in the formation of the internal aldimine with PLP. DHPAA-S, NCBI protein ID: EAT37246 or VectorBase gene ID: AAEL010735; Ddc, NCBI protein ID: EAT33489 or VectorBase gene ID: AAEL014238. 40 carboxyl side residues, present in DHPAA synthase, but absence in Ddc, were not included in the alignment.
(DOC)

Figure S2 Sequence comparison of different mosquito DHPAA synthases. Sequences A and B are reassembled full-length *Anopheles gambiae* (XP_319838) and *Culex quinquefasciatus* DHPAA synthases, respectively. Sequence alignment C illustrates high conservation of DHPAA synthases from three mosquito species. Residues in blue (in A & B) are those missed in the database. The N-terminal fragment is essential for DHPAA synthase activity, because a *Cu. quinquefasciatus* recombinant protein, initially expressed without the N-terminal fragment (the first 43 residues in blue), was inactive. Active recombinant *Cu. quinquefasciatus* DHPAA synthase was obtained after the N-terminal fragment was included.
(DOC)

Table S1 Oligonucleotide primers for recombinant DHPAA synthase protein expression. Oligonucleotide primer pairs were synthesized based on the coding sequences of individual *Drosophila* and mosquito DHPAA synthases and used for amplification of their corresponding cDNA sequences. The underlined nucleotides represent the introduced restriction sites.
(DOC)

## References

[1] Sheng J, An K, Deng C, Li W, Bao X (2006). Cloning a cuticle-degrading serine protease gene with biologic control function from Beauveria brongniartii and its expression in Escherichia coli.. Curr Microbiol.

[2] Luz C, Batagin I (2005). Potential of oil-based formulations of Beauveria bassiana to control Triatoma infestans.. Mycopathologia.

[3] Andersen SO (2001). Matrix proteins from insect pliable cuticles: are they flexible and easily deformed?. Insect Biochem Mol Biol.

[4] Andersen SO, Hojrup P, Roepstorff P (1995). Insect cuticular proteins.. Insect Biochem Mol Biol.

[5] Andersen SO (2010). Insect cuticular sclerotization.. Insect Biochem Mol Biol.

[6] Andersen SO, Gilbert LI, Iatrou K, Gill SS (2005). Cuticular sclerotization and tanning.. Comprehensive Molecular Insect Science.

[7] Nene V, Wortman JR, Lawson D, Haas B, Kodira C (2007). Genome sequence of *Aedes aegypti*, a major arbovirus vector.. Science.

[8] Wright TR, Bewley GC, Sherald AF (1976). The genetics of dopa decarboxylase in *Drosophila melanogaster*. II. Isolation and characterization of dopa-decarboxylase-deficient mutants and their relationship to the alpha-methyl-dopa-hypersensitive mutants.. Genetics.

[9] Wright TR, Hodgetts RB, Sherald AF (1976). The genetics of dopa decarboxylase in *Drosophila melanogaster*. I. Isolation and characterization of deficiencies that delete the dopa-decarboxylase-dosage-sensitive region and the alpha-methyl-dopa-hypersensitive locus.. Genetics.

[10] Sherald AF, Wright TR (1974). The analog inhibitor, alpha-methyl dopa, as a screening agent for mutants elevating levels of dopa decarboxylase activity in *Drosophila melanogaster*.. Mol Gen Genet.

[11] Marsh JL, Wright TR (1986). Evidence for regulatory variants of the dopa decarboxylase and alpha-methyldopa hypersensitive loci in *Drosophila*.. Genetics.

[12] Sparrow JC, Wright TR (1974). The selection for mutants in *Drosophila melanogaster* hypersensitive to alpha-methyl dopa, a dopa decarboxylase inhibitor.. Mol Gen Genet.

[13] Black BC, Pentz ES, Wright TR (1987). The alpha methyl dopa hypersensitive gene, 1(2)amd, and two adjacent genes in *Drosophila melanogaster*: physical location and direct effects of amd on catecholamine metabolism.. Mol Gen Genet.

[14] Marsh JL, Erfle MP, Leeds CA (1986). Molecular localization, developmental expression and nucleotide sequence of the alpha-methyldopa hypersensitive gene of *Drosophila*.. Genetics.

[15] Wang D, Marsh JL (1995). Developmental regulation of the alpha-methyldopa hypersensitive gene of Drosophila melanogaster.. Dev Biol.

[16] Wang D, Marsh JL, Ayala FJ (1996). Evolutionary changes in the expression pattern of a developmentally essential gene in three Drosophila species.. Proc Natl Acad Sci U S A.

[17] Rees JN, Florang VR, Eckert LL, Doorn JA (2009). Protein reactivity of 3,4-dihydroxyphenylacetaldehyde, a toxic dopamine metabolite, is dependent on both the aldehyde and the catechol.. Chem Res Toxicol.

[18] Marchitti SA, Deitrich RA, Vasiliou V (2007). Neurotoxicity and metabolism of the catecholamine-derived 3,4-dihydroxyphenylacetaldehyde and 3,4-dihydroxyphenyl glycolaldehyde: the role of aldehyde dehydrogenase.. Pharmacol Rev.

[19] Bortolato M, Chen K, Shih JC (2008). Monoamine oxidase inactivation: from pathophysiology to therapeutics.. Adv Drug Deliv Rev.

[20] Cutillas B, Ambrosio S, Unzeta M (2002). Neuroprotective effect of the monoamine oxidase inhibitor PF 9601N [N-(2-propynyl)-2-(5-benzyloxy-indolyl) methylamine] on rat nigral neurons after 6-hydroxydopamine-striatal lesion.. Neurosci Lett.

[21] Burke WJ, Li SW, Chung HD, Ruggiero DA, Kristal BS (2004). Neurotoxicity of MAO metabolites of catecholamine neurotransmitters: role in neurodegenerative diseases.. Neurotoxicology.

[22] Meyer JH, Ginovart N, Boovaruwala A, Sagrati S, Hussey D (2006). Elevated monoamine oxidase a levels in the brain: an explanation for the monoamine imbalance of major depression.. Arch Gen Psychiatry.

[23] Youdim MB, Bakhle YS (2006). Monoamine oxidase: isoforms and inhibitors in Parkinson's disease and depressive illness.. Br J Pharmacol.

[24] Petzer JP, Castagnoli N, Schwarzschild MA, Chen JF, Van der Schyf CJ (2009). Dual-target-directed drugs that block monoamine oxidase B and adenosine A(2A) receptors for Parkinson's disease.. Neurotherapeutics.

[25] Dunkel P, Gelain A, Barlocco D, Haider N, Gyires K (2008). Semicarbazide-sensitive amine oxidase/vascular adhesion protein 1: recent developments concerning substrates and inhibitors of a promising therapeutic target.. Curr Med Chem.

[26] Saunders EF, Silk KR (2009). Personality trait dimensions and the pharmacological treatment of borderline personality disorder.. J Clin Psychopharmacol.

[27] Youdim MB (2006). The path from anti Parkinson drug selegiline and rasagiline to multifunctional neuroprotective anti Alzheimer drugs ladostigil and m30.. Curr Alzheimer Res.

[28] Han Q, Ding H, Robinson H, Christensen BM, Li J (2010). Crystal structure and substrate specificity of *Drosophila* 3,4-dihydroxyphenylalanine decarboxylase..

[29] Loutelier-Bourhis C, Legros H, Bonnet JJ, Costentin J, Lange CM (2004). Gas chromatography/mass spectrometric identification of dopamergic metabolites in striata of rats treated with L-DOPA.. Rapid Commun Mass Spectrom.

[30] Mattammal MB, Chung HD, Strong R, Hsu FF (1993). Confirmation of a dopamine metabolite in parkinsonian brain tissue by gas chromatography-mass spectrometry.. J Chromatogr.

[31] Slates HL, Taub D, Kuo CH, Wendler NL (1964). Degradation of α-Methyl-3,4-dihydroxyphenylalanine (α-MethylDOPA).. J Org Chem.

