# Supplementary material for: From L-Dopa to Dihydroxyphenylacetaldehyde: A Toxic Biochemical Pathway Plays a Vital Physiological Function in Insects
Source: PLoS One. 2011 Jan 24;6(1):e16124. doi: 10.1371/journal.pone.0016124 (PMC3026038; doi:10.1371/journal.pone.0016124)
Supplement: Figure S1 — Sequence comparison of Ae. aegypti DHPAA synthase and Ddc. Letters with magenta and cyan background are fully conserved and strongly conserved amino acid residues, respectively. Lys306 in DHPAA-S and Lys303 (yellow background) in Ddc are involved in the formation of the internal aldimine with PLP. DHPAA-S, NCBI protein ID: EAT37246 or VectorBase gene ID: AAEL010735; Ddc, NCBI protein ID: EAT33489 or VectorBase gene ID: AAEL014238. 40 carboxyl side residues, present in DHPAA synthase, but absence in Ddc, were not included in the alignment. (DOC) [file pone.0016124.s001.doc]

DHPAA-S MANMDIDEFKEFGKAAIDFVADYLVNIRDRDVLPSVEPGYLHDLLPNEIPEKGDDWKTIMEEFKRFIVPG

DDC ---MQAPQFKDFAKEMVDYIANYLENIRDRRVLPEVQPGYLKPLIPSEAPEKPESWEAVMADIERVIMPG

DHPAA-S LTHWQSPHFHAFYPSQTSYSSIVGETLAAGLGVVGFSWICSPVCTELEVIMMNWIGQLLNLPRCFLNCDE

DDC VTHWHSPKFHAYFPTANSYPAIVADMLSGAIACIGFTWIASPACTELEVEMLNWLGKMLGLPEEFLASSG

DHPAA-S GNGGGVIQGSASESIFIAVLVAREQAVRRLKNEHPELTEAEIRGRLVAYTSDQSNSAVEKSGILGAIKMR

Ddc GQAGGVIQGTASEATLVALLGAKAKAIKRTQEEHPEWDETYIISRLVGYTSNQSHSSVERAGLLGGVKLR

DHPAA-S LLPADDDCVLRGRTLKKAVEEDKAYGLFPVIMVATLGTTGTCAYDNLEEIGPYCNDNKLWLHVDAAYAGA

DDC SLKADSNLQLRGETLEEAIKQDLADGLIPFYAVCTLGTTNTCAFDRLDELGPVGNKYNVWIHVDAAYAGS

DHPAA-S SFCLPEYAWIKKGLEMADSLNFNLH**K**WLFVNFDCCAMWFKDAAMITEAFSVDRIYLQHKFQGMSKAPDYR

DDC AFVCPEYRHLMKGIETADSFNFNPH**K**WMLVNFDCSAMWLKEPYWIVNAFNVDPLYLKHDMQGS--APDYR

DHPAA-S HWQIQLGRRFRSLKVWITLKTMGAEKIRELIRFHISLAQKFEQYVRADPRFEVTSST-LALVCFRLKG E

Ddc HWQIPLGRRFRALKLWFVLRLYGVENIQAHIRRHCAFAKQFEALCVADSR FEIFSTVQMGLVCFRLKGN

DHPAA-S DTYSKQLLDNIVKRKKIYMIPATYQGKFILRFMIAGIDPQAEDIDYAWNEVKSQTDLLLGV

DDC NEISEALLKKINGRGKIHMVPSKVNDVYFLRMAVCSRFTEASDIEYSWNEVSAVADELLAE

Figure S1. Sequence comparison of *Ae. aegypti* DHPAA synthase and Ddc. Letters with magenta and cyan background are fully conserved and strongly conserved amino acid residues, respectively. Lys306 in DHPAA-S and Lys303 (yellow background) in Ddc are involved in the formation of the internal aldimine with PLP. DHPAA-S, NCBI protein ID: EAT37246 or VectorBase gene ID: AAEL010735; Ddc, NCBI protein ID: EAT33489 or VectorBase gene ID: AAEL014238. 40 carboxyl side residues, present in DHPAA synthase but absence in Ddc, were not included in the alignment.
