# Supplementary material for: From L-Dopa to Dihydroxyphenylacetaldehyde: A Toxic Biochemical Pathway Plays a Vital Physiological Function in Insects
Source: PLoS One. 2011 Jan 24;6(1):e16124. doi: 10.1371/journal.pone.0016124 (PMC3026038; doi:10.1371/journal.pone.0016124)
Supplement: Figure S2 — Sequence comparison of different mosquito DHPAA synthases. Sequences A and B are reassembled full-length Anopheles gambiae (XP_319838) and Culex quinquefasciatus DHPAA synthases, respectively. Sequence alignment C illustrates high conservation of DHPAA synthases from three mosquito species. Residues in blue (in A & B) are those missed in the database. The N-terminal fragment is essential for DHPAA synthase activity, because a Cu. quinquefasciatus recombinant protein, initially expressed without the N-terminal fragment (the first 43 residues in blue), was inactive. Active recombinant Cu. quinquefasciatus DHPAA synthase was obtained after the N-terminal fragment was included. (DOC) [file pone.0016124.s002.doc]

A

>Anopheles gambiae XP_319838

MANMDINEFREFGRAAIDFVADYLENIRDRDVLPSVEPGYLHDLLPGELQNEPEDWKTIMEDFKRCILPGLTHWQSPHFH AFYPSQTSYSSIVGETLAAGLGVVGFSWVCSPVCTELEVIMMNWLGQLLNLPKSFLNCDEGNGGGIIQGSASESILVAVL AAREQAVRRLRTQHPELTEADIRGRLVAYTSDQSNSAVEKSGILGAIKMRLLPADETGILRGSTFIQAVEEDVAKGLFPV ICVATLGTTGTCAYDNLAEIGPYCNEHNIWLHIDAAYAGAALCLPEYTHIMKGAELADSLNFNLHKWMFVNFDCCAMWFK DAGSVTQSFSVDRIYLQHQFQGHSKAPDYRHWQIQLGRRFRSLKVWITLRTMGAEKIRSLIRFHIQLANRFEEYVRTDDR FEVLCSTLALVCFRLKGDDARSKQLLENITKRKKIFMIPATYQGKFIIRFMICGIDPQMHDIEYAWEEVRSQADLLLGVD

ENRNVAKQAIEPVEPKLYEKSSEIGKITESLTGMVISSEKGQ

B

>Culex quinquefasciatus EDS39185

MANMDVNEFREFGKAAIDWVADYLENVRDREVLPSVEPGYLHNMIPSEIPEHGDHWKSIMEDFKRCILPGITHWQSPNFH AFYPSQTSYSSIVGETLAAGLGVVGFSWICSPACTELEVIMMNWLGQLLNLPKCFLNCDDGNGGGIIQGSASESIFVAVL VAREQAVRRLKVEHPELTEAEIRGRMVAYTSDQSNSAVEKSGILGAIKMRLLPANDDCVLRGSTLIKAVEEDKANGLFPV IMVATLGTTGTCAYDKLEEIGPYCNENNIWLHIDAAYAGASFCLPEYAWIKKGLEAADSLNFNLHKWLFVNFDCCAMWFK DANRITDAFSVDRIYLQHKYQGQSKAPDYRHWQIQLGRRFRSLKVWVTLKTMGAEKIRELIRFHISLAQKLEAYARADDR FEVTSSTLALVCFRLKGEDALSKQLLENITKRKKIYMIPATFQGKFILRFMIGGIDPQPADIDYAWNEIKTQTDVLLGVD

ENGNDVTVKDIIKQEMFEKEKPIGMITESLNGLVLANEKAK

C


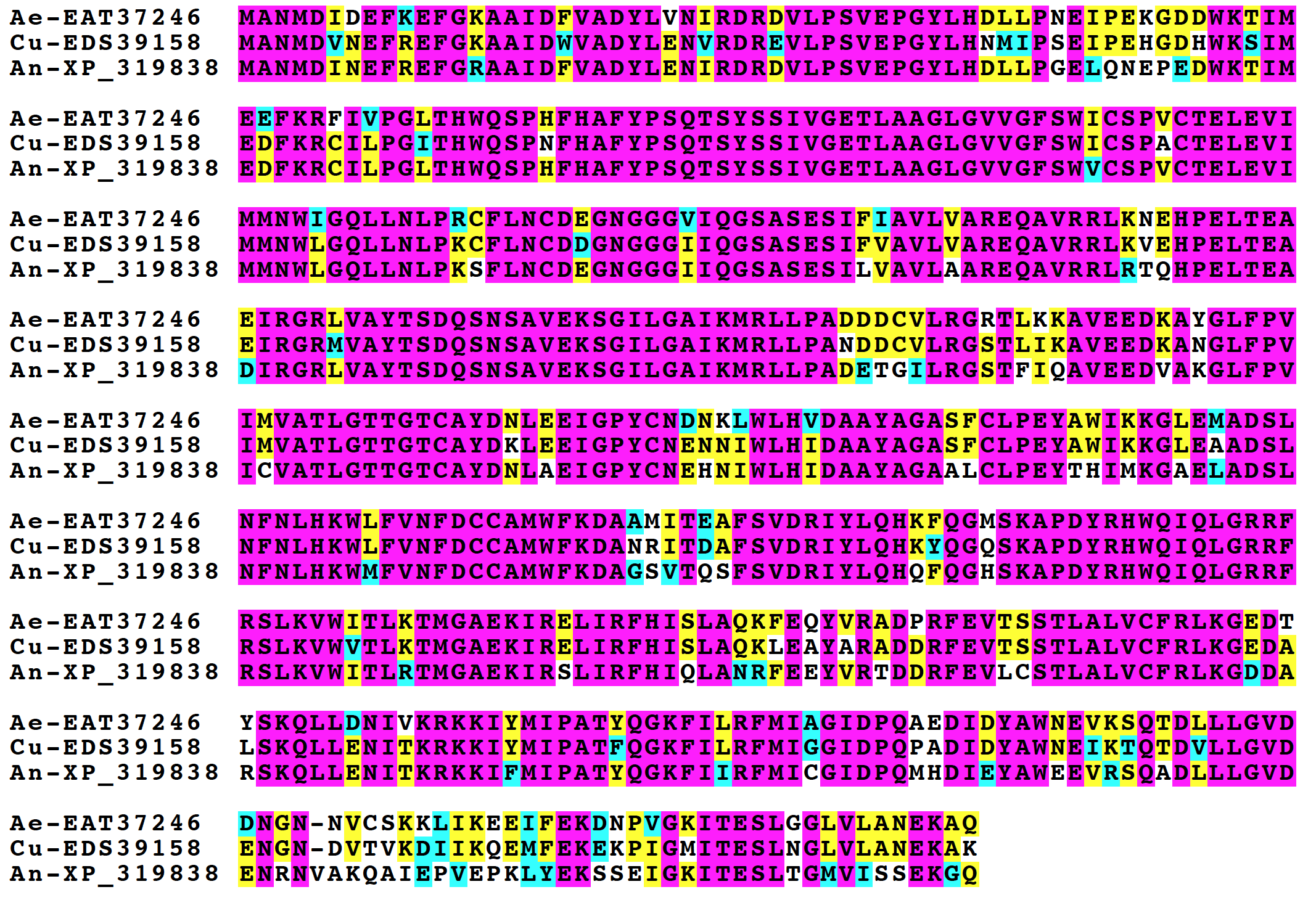


Figure S2. DHPAA synthase sequences from *Anopheles gambiae* (XP_319838) (A) and *Culex quinquefasciatus* (EDS39185) (B) and their sequence alignment with *Aedes aegypti* DHPAA synthase (EAT37246) (C). Residues in blue (in A & B) are those missed in the database. The N-terminal fragment is essential for DHPAA synthase activity, because a *Culex quinquefasciatus* recombinant protein, initially expressed without the N-terminal fragment (the first 43 residues in blue), was inactive. Active recombinant *Cu. quinquefasciatus* DHPAA synthase was obtained after the N-terminal fragment was included.
