# Supplementary material for: From L-Dopa to Dihydroxyphenylacetaldehyde: A Toxic Biochemical Pathway Plays a Vital Physiological Function in Insects
Source: PLoS One. 2011 Jan 24;6(1):e16124. doi: 10.1371/journal.pone.0016124 (PMC3026038; doi:10.1371/journal.pone.0016124)
Supplement: Table S1 — Oligonucleotide primers for recombinant DHPAA synthase protein expression. Oligonucleotide primer pairs were synthesized based on the coding sequences of individual Drosophila and mosquito DHPAA synthases and used for amplification of their corresponding cDNA sequences. The underlined nucleotides represent the introduced restriction sites. (DOC) [file pone.0016124.s003.doc]

Table S1. Oligonucleotide primers for recombinant DHPAA synthase protein expression.

| Protein ID | Primers | Nucleotide sequences | Restriction site |
| --- | --- | --- | --- |
| EAT37246 | Forward  Reverse | AAAAGAATGCTATGGCGAATATGGATATAGATGAGTT  AAAAGAATTCACTGAGCTTTTTCGTTGGCAAGCA | Bsm I  EcoR I |
| NP_476592  Isoform A | Forward  Reverse | AAAACATATGATGGATGCCAAGGAGTTTCGGGAAT  AAAACTCGAGTCACTGAGATTTCTCGTGCGTTG | Nde I  Xho I |
| NP_724162  Isoform B | Forward  Reverse | AAAAGAATGCTATGGACTTTGATGAGTTCCGTG  AAAACTCGAGTCACTGAGATTTCTCGTGCGTTG | Bsm I  Xho I |
| XP_319838 | Forward  Reverse | AAAACATATGACGTCCTACTCATCGATCGTG  AAAAGAATTCAATGTTCTCCAGCAGCTGCT | Nde I  EcoR I |
| XP_319838* | Forward  Reverse | AAAACATATGGCAAACATGGACATTAATGA  AAAAGAATTCACTGTCCCTTTTCGCTCGAAATTAC | Nde I  EcoR I |
| EDS39158 | Forward  Reverse | AAAACATATGATCCCGTCTGAGATTCCC  AAAAGAATTCACTTTGCCTTTTCATTAGCCAA | Nde I  EcoR I |
| EDS39158* | Forward  Reverse | AAAACATATGGCGAATATGGACGTTAACGAGTT  AAAAGAATTCACTTTGCCTTTTCATTAGCCAACAC | Nde I  EcoR I |

Oligonucleotide primer pairs were synthesized based on the coding sequences of individual *Drosophila* and mosquito DHPAA synthases and used for amplification of their corresponding coding sequences. The underlined nucleotides represent the introduced restriction sites. Protein ID for DHPAA synthases: EAT37246 from *Aedes aegypti*; NP_476592 Isoform A and NP_724162 Isoform B from *Drosophila melanogaster*; XP_319838 from *Anopheles gambiae*; EDS39158 from *Culex quinquefasciatus*. Recombinant proteins, expressed from reported CDS of *Anopheles gambiae* XP_319838 and *Culex quinquefasciatus* EDS39158 in protein databases, showed no DHPAA activity. Further analysis indicated that *An. gambiae* XP_319838 is missing a 5’-end exon and a 3’-end exon and the CDS of the *Cu. quinquefasciatus* EDS39158 is missing a 5’-end exon (see Figure 3S). When their full-length CDS were amplified (with primer pairs of XP_319838* and EDS39158*, respectively) and expressed, their recombinant proteins are biochemically active as *Aedes aegypti* and *D. melanogaster* DHPAA synthases.
